# Interdisciplinary Overview of Lipopeptide and Protein-Containing Biosurfactants

**DOI:** 10.3390/genes14010076

**Published:** 2022-12-26

**Authors:** Régis Antonioli Júnior, Joice de Faria Poloni, Éderson Sales Moreira Pinto, Márcio Dorn

**Affiliations:** 1 Center for Biotechnology, Federal University of Rio Grande do Sul, Porto Alegre 91501-970, Brazil; 2 School of Health and Life Sciences, Pontifical Catholic University of Rio Grande do Sul, Porto Alegre 90619-900, Brazil; 3 National Institute of Science and Technology—Forensic Science, Porto Alegre 90619-900, Brazil; 4 Institute of Informatics, Federal University of Rio Grande do Sul, Porto Alegre 91501-970, Brazil

**Keywords:** biosurfactant, lipopeptide, protein-containing biosurfactant, bioinformatic, biosurfactant physicochemical properties

## Abstract

Biosurfactants are amphipathic molecules capable of lowering interfacial and superficial tensions. Produced by living organisms, these compounds act the same as chemical surfactants but with a series of improvements, the most notable being biodegradability. Biosurfactants have a wide diversity of categories. Within these, lipopeptides are some of the more abundant and widely known. Protein-containing biosurfactants are much less studied and could be an interesting and valuable alternative. The harsh temperature, pH, and salinity conditions that target organisms can sustain need to be understood for better implementation. Here, we will explore biotechnological applications via lipopeptide and protein-containing biosurfactants. Also, we discuss their natural role and the organisms that produce them, taking a glimpse into the possibilities of research via meta-omics and machine learning.

## 1. Introduction

Biosurfactants are amphipathic molecules synthesized by plants, animals, and microbes, that reduce interfacial and superficial tensions in aqueous solutions and hydrocarbon mixtures [1]. Due to these properties, biosurfactants alter how other molecules interact, increasing the solubility of substances [2,3]. The hydrophilic portion—the head—will usually be a hydrocarbon, while the hydrophobic portion—the tail—can be non-ionic, positively or negatively charged, or amphoteric [4]. These characteristics allow biosurfactants to form aggregates called micelles, which gather when there is an increase in amphiphilic concentration in a liquid beyond a limit, known as critical micelle concentration (CMC) [5].

Most chemically produced surfactants are petroleum-based, and the hydrophobic parts consist of paraffins, olefins, alkylbenzenes, alkylphenols, and alcohols and the hydrophilic domain is usually a sulfate, or a sulphonate [6,7,8]. Some examples of widely used surfactants include sodium n-dodecyl sulfate, and Triton X100 [2]. They pose occupational and environmental risks, as surfactants can be extremely dangerous to the environment if not carefully applied. For example, the use of dispersants, which are a type of chemical surfactant, during oil spills has a series of documented issues and concerns [9,10]. Some studies even described dispersants being capable of diminishing microbial degradation activity [11,12]. Therefore, there is a concern regarding their usage, and research to find less aggressive alternatives is growing [13].

In contrast to the artificial surfactants, biosurfactants are eco-friendly alternatives that offer many advantages when compared to synthetic surfactants, such as surface and interfacial activity, resistance to temperature, pH and ionic force, low toxicity, availability, specificity, biocompatibility, and biodegradability [14,15,16]. Due to their varied structural diversity and composition, the biosurfactants themselves are used in various applications. Applicable areas include bioremediation, medicine, food industry, and industrial processes [17]. Though biosurfactants possess valuable capabilities, large-scale industrial applications are hindered by high costs and low efficiency of their production and recovery processes [13]. Nevertheless, the demand for biosurfactants is increasing, and both the industry and consumers are willing to pay for an alternative that is more beneficial in the long run [18]. Several strategies for optimal growth and production have been researched over the years, and this scenario is gradually changing [3,19,20,21].

There are several classes of compounds among biosurfactants, as they have a vast structural diversity. They can be categorized as glycolipids, lipopeptides and lipoproteins, fatty acids, phospholipids, natural lipids, polymeric and particulate [22,23,24,25].

Regarding lipopeptides and lipoprotein biosurfactants, the leading producers are fungi, bacteria, and yeast [26] They act to enhance mobility, decrease viscosity, facilitate solubilization, and act as metal-sequestering agents [26]. Additionally, they can disrupt biological membranes, making them potential agents to be used as hemolytic, antiviral, antibacterial, and anti-carcinogenic molecules [26]. One of the main interests in biosurfactant research is employing them as surface-active compounds in bioremediation strategies as alternatives for traditional chemical methods. This review aims to present what is known about these proteic compounds, be it how they provide advantages to their producers, their potential for biotechnological applications, and how novel biosurfactants can be discovered and produced. While also going over the different production techniques and novel research methods using bioinformatic approaches such as omics technology, molecular dynamics, and machine learning.

## 2. Biosurfactant-Producing Microorganisms

Microorganisms like bacteria, filamentous fungi, and yeasts can synthesize biosurfactants of varied molecular structures and surface properties [27,28,29]. Two of the most researched biosurfactant-producing microorganisms are the *Pseudomonas* and *Bacillus* genres. Table 1 shows a list of some microorganisms of interest and their produced biosurfactants. Some studies on the potential of lipoproteins in facilitating microbial hydrocarbon degradation have been conducted [30,31,32,33]. Interestingly to note is the study in which a hydrocarbon-degrading consortium of microorganisms was constructed with samples collected from uncontaminated soil [30]. It highlights the importance of how microbial interaction between different species might dictate the fate of pollutant degradation and offers new insights into bioaugmentation applications.

Akin to other surface-active molecules, biosurfactants contain one or several lipophilic and hydrophilic moieties. Biosurfactants are categorized by their chemical composition, mechanism of action, molecular weight, physicochemical properties, and microbial origin [28,34]. According to their molecular weight, biosurfactants can be divided into low molecular weight compounds that include phospholipids, glycolipids, and lipopeptides and into high molecular weight that contains amphipathic polysaccharides, lipoproteins, lipopolysaccharides, or more complex mixtures of those [24,25,28]. The low molecular weight molecules, such as rhamnolipids produced by *Pseudomonas* efficiently lower surface tension and interfacial tension, while high molecular weight polymers, like the surfactin produced by *Bacillus subtilis*, bind tightly to surfaces [35]. We will focus on the peculiarities of the lipopeptide and protein-containing biosurfactants. The lipophilic moiety is a protein or a peptide with hydrophobic side chains or a hydrocarbon chain of a fatty acid. The hydrophilic moiety is an ester, a hydroxy, phosphate, or carboxylate group, or a sugar carbohydrate [28]. In addition, there are highly surface-active globular proteins, such as hydrophobins, an amphiphilic protein that will bediscussed later.

**Table 1:** Properties and characteristics of the main lipopeptide and protein-containing biosurfactants.

Biosurfactant/Bioemulsifier	Class	Producing Species	Reported Genes	Ionic Charge	Molecular Weigth (KDa)	CMC (mg/L)	Superficial Tension (mN/m)	Potential Application	Ref.
Alasan	Polymeric	*Acinetobacter calcoaceticusAcinetobacter radioresistens*	*aln A-C*	Negative	1000	*-*	41.6	Emulsification, solubilization activity	[36,37,38,39,40]
Hydrophobin	Globular protein	*Lecanicillium lecaniiTrichoderma reeseiSchizophyllum commune*	*HFBI, HFBII*	*-*	<20	*-*	25–45	Drug solubilization biomineralization	[41,42]
Arthrofactin	Lipopeptide	*Pseudomonas* sp.	*arfA–C*	-	1.354	13.5	72–24	Antifungal	[43,44]
Fengycins	Lipopeptide	*B. subtilis*	*fenA–E* gene cluster	Negative	1.463	-	21	Antifungal, antimicrobial	[45,46,47,48]
Iturin	Lipopeptide	*B. subtilisBacillus pumilus*	*ituA–C*	Neutral	1.043	*-*	30–37.5	Antimicrobial, biopesticides	[36,40,49,50]
Lichenysin	Lipopeptide	*Bacillus licheniformisB. subtilis*	*licA–D*	Negative	0.993–1.049	10–22	27	Oil recovery, hemolytic, chelating agent	[40,51,52,53]
Serrawettin	Lipopeptide	*Serratia marcescensSerratia surfactantfaciens*	*pswP*	Neutral	0.541–0.731	*-*	28–33.9	Oil recovery, antimicrobial, antitumoral	[4,40,49,54,55]
Surfactin	Lipopeptide	*B. subtilis*	*srfA–D*	Negative	1.007–1.035	20–40	22–27.9	Oil recovery, antibacterial, antitumoral, antiviral, anticoagulant	[4,36,40,52]
Syringomycin	Lipopeptide	*Pseudomonas syringae* B301D	*syrB1*, *syrE*, *syrB2*, *syrC*, *syrP*	Positive	1.225	1250	33	Antibacterial	[43,56,57]
Syringopeptin	Lipopeptide	*P. syringae* B301D	*sypA–C*	Positive	2.399	820	40.2	Antibacterial	[43,56,57,58]
Viscosin	Lipopeptide	*Pseudomonas fluorescens*	*viscA–C*	*-*	1.126	10–15	26.5–28	Antitumoral, antibacterial	[59,60,61]

### 2.1. Lipopeptides

**Figure 1:**
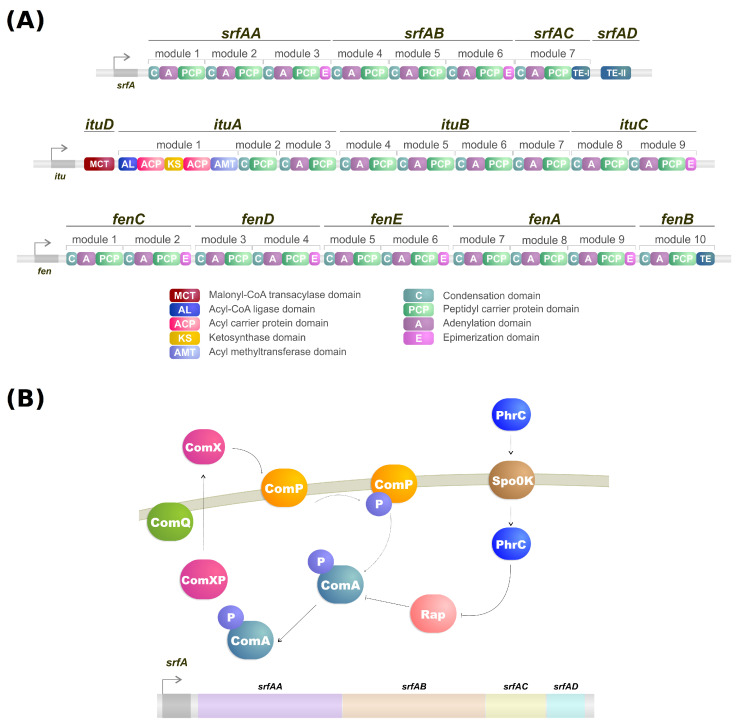
(**A**) NRPS modules of surfactin, iturin, and fengycin. (**B**) Simplified model of transcriptional regulation of *srfA* gene from *Bacillus*.

Lipopeptides are a class of biosurfactants with high industrial interest. They are linear or cyclic oligopeptides acylated with fatty acids of different length and composition which are synthesized by the nonribosomal peptide synthetases (NRPSs) [62] and commonly secreted by bacterial genres such as *Bacillus*, *Streptomyces* and *Pseudomonas* [25]. Microorganisms of the genus *Bacillus* are some of the more well-known lipopeptide producers, and their genome contains a large operon (srfA) composed of four open reading frames which encode a peptide synthetase responsible for the surfactin biosynthesis (Figure 1A,B) [63,64]. Lipopeptides have varying emulsification properties according to temperature, pressure, pH, structure and stability of the solution [65]. For example, the emulsifying activity of surfactin produced by *B. subtilis* can change based on pH. With a pH above 7, it forms a stable emulsion with kerosene, but when the pH drops to 3 the emulsion does not form [66].

The lipopeptidic biosurfactants offer a great range of natural advantages for their microbial producers. Some noteworthy examples include the secretion of polymers aiding in the structure of biofilm formation and, in turn, improving survival against adverse conditions such as antibiotic treatment and shearing [67]. Moreover, predator avoidance is another significant natural function of biosurfactants.Laboratory studies showed that the serrawetin W2 produced by *S. marcescens* and surfactin produced by *B. subtilis* induced *Caenorhabditis elegans* individuals to avoid them [68]. Lipopeptides were also shown to affect interaction in natural habitats, in vitro testing with lipopeptides produced by both *Pseudomonas* and *Bacillus* species exhibited lytic activity and inhibited growth against microorganisms such as viruses, mycoplasmas, bacteria, fungi, and oomycetes [62,69,70].

For commercial interests, biosurfactants must have good yield levels and cost of production. In this sense, surfactin was reported to have increased yield (36-fold), reaching 3.6 g/L, by *B. subtilis* ATCC 21332 when incorporated with activated carbon [71]. Compared to some other types of biosurfactants, such as the glycolipids (i.e., rhamnolipids), that reach yields of 1 g/L, surfactin shows many prospects for industrial use [63]. Another study investigated the bioremediation potential of surfactin produced by *B. subtilis* ATCC 21332, and compared it with rhamnolipid produced by *P. aeruginosa* J4 for enhanced degradation of diesel-contaminated water and soil, and showed that both had successfully reached degradation levels between 80-100% and CMCs between 45 mg/L and 50 mg/L, further confirming surfactin as an efficient lipopeptide biosurfactant [72].

### 2.2. Protein-Containing Biosurfactants

Surface-active proteins are a lesser-studied class of biosurfactants that still has some relevance for industrial applications. Depending on the author, lipopeptides will occasionally be grouped with lipoproteins [73]. Considered as high molecular mass bioemulsifiers, they are complex structures with multiple reactive groups exposed, turning them into effective emulsifiers as they bind tightly to hydrophobic molecules [63]. Surface tension at liquid-air interfaces is a significant barrier encountered by distinct organisms. Conquering the surface tension is an essential mechanism in several species, i.e., as sporulation of bacteria and fungi, foaming in frog nests, and evaporative cooling in horses [25,73]. These biosurfactants are divided into different groups according to structure: lipid-associated proteins, such as pulmonary surfactants [74], and non-lipid-associated globular proteins, such as hydrophobins [73]. Polymeric biosurfactants are a different category of high molecular weight biosurfactants containing protein in their composition [26]. Alasan (Table 1) is the best-known biopolymer of this group and consists of a high molecular weight polysaccharide and protein complex with solubilizing and emulsifying activity [26]. Another effective emulsifier is liposan, composed of 87% carbohydrates and 17% proteins, produced by *Candida lipolytica* [26,75]. It was first described in 1985 by Cirigliano and Carman [75], who showed that it is not able to reduce the surface tension of water but effectively emulsified and stabilized water-in-oil emulsion [26]. There are still few studies that explore the functionality and application of these biopolymers.

The bacterial species *B. subtilis* has been shown to protect plant roots from pathogenic fungi and bacteria by using lipoproteins [73]. Upon colonizing the area and forming a biofilm, it produces the lipoprotein BslA on the outer layer, thus forming a hydrophobic barrier, often called a “bacterial raincoat” [73,76,77]. This is the more common natural function of the globular biosurfactants, as other examples include the hydrophobins produced by many fungi to create a hydrophobic layer on the hydrophilic hypha or spores for protection [73,78,79,80,81,82]. Moreover, surface-active proteins have some described biotechnological applications, such as using their properties of self-assembly and reversal of surface wettability in nanodevice and medical implant coating, e.g., coating electrodes and the surface of biliary stents to prevent oxidation and fouling, respectively [73,83].

## 3. Lipopeptides Synthesis and Regulation

Biosurfactants are secondary metabolites produced by microorganisms, and as commented in a previous section, the lipopeptides, specifically, are produced by NRPSs.

NRPSs are multidomain mega-enzymes that synthesize NRPs without using the ribosomal machinery [84]. They have a modular structure in which each part incorporates an amino acid to the peptide moiety of the lipopeptide (Figure 1B). Another characteristic is that the NRPS obeys the colinearity rule; that is, the modules are colinear with the amino acid sequence of the peptide [62]. There are two types of modules: initiation and elongation modules. Each of these modules also contains domains that perform specific tasks [62]. While typically, initiation modules have domains responsible for the amino acid election, activation, and thioesterification of the activated amino acid, the first module also contains a condensation domain in lipopeptide biosynthesis. This domain is responsible for catalyzing the *N*-acylation of the first amino acid of the lipopeptide, thus linking the lipid moiety to the oligopeptide [62,85]. Elongation modules contain the same domains, but this time the condensation domain is responsible for catalyzing the peptide bond between two amino acids. The condensation domain from the elongation module will generate a lipopeptide that, by the end of the assembly line, will be cleaved by a thioesterase [62].

It has been shown via analyses of the metabolic profiles of *Pseudomonas* and *Bacillus* species that a single strain may be able to produce different forms of the same biosurfactant [62]. Examples include *B. subtilis* strain OKB 105, which can produce 12 different surfactin analogs [86] and *P. fluorescens* strain SS101 that can produce up to eight analogs of the lipopeptide massetolide A [87]. These analogs are thought to be the product of flexibility in the amino acid selection and activation by the adenylation domain of the initiation module in NRPSs. Said flexibility is common in nonribosomal peptide synthesis, which may have biological purposes for the producers [62].

Several advancements have been made in the quest to elucidate biosurfactant synthesis regulation over the decades. It is known that, for example, in *Pseudomonas*, the GacA/GacS regulatory system is the primary regulator in lipopeptide production. A mutation in either one of the encoding genes results in the loss of this function [88,89,90].

Iturin production has a series of regulation factors. For example, the methylation of tyrosine residues in iturin can decerease yield and antibacterial activity [91,92]. Furthermore, iturin can also be regulated by controlling the expression of sigma factor A and the transcription factor ComA. Overexpressing the genes *sigA* and *comA* increased iturin yield [93].

As mentioned, the biosynthesis of surfactin is coordinated by the *srfA* operon, which contains four open reading frames (*srfAA*, *srfAB*, *srfAC*, and *srfAD*) as seen in Figure 1A [94]. These open reading frames are responsible for the peptide chain extension, a key step in the surfactin synthesis regulation [92]. Surfactin, much like iturin, is also strongly regulated by the transcription factor ComA, which binds to *srfA*, and thus controls its transcription and regulates *srfA* expression (Figure 1B) [92]. ComA phosphorylation is the key to activating the *srfA* operon transcription by two different pathways. The first involves the ComX peptide modified by ComQ that stimulates ComP autophosphorylation, triggering ComA phosphorylation (Figure 1A) [43]. Activated ComA translocates to the nucleus and promotes *srfA* transcription. PhrC importation is mediated by Spo0K, which interacts with Rap protein and inhibits its phosphatase activity, thus, preventing ComA dephosphorylation and facilitating ComA-induced *srfA* transcription (Figure 1A) [43,95].

Another regulatory system tightly related to biosurfactant production is quorum sensing, identified in a series of lipopeptide-producing species. Quorum sensing is a cell-to-cell communication process where colonies of bacteria identify population densities via detecting secreted molecules called autoinducers, tracking cell density, and adjusting gene regulation accordingly [96,97,98]. Examples include the production of serrawetin W2 in *Serratia liquefaciens*, with the quorum sensing genes *swrI*-*swrR* and regulated by N-butanoyl-L-homoserine lactone and N-hexanoyl-L-homoserine lactone production [99], and the production of surfactin in *B. subtilis* via the *comA*-*comP* genes which is regulated by competence and sporulation stimulating factor [100].

It is known that the pathways of different lipopeptides iteract with each other. For example, knocking out the *sfp* gene can significantly increase plipastasin production, while regulating plipastasin also increases the production of surfactin [101,102]. On the other hand, the absence of *srfAB* lowers surfactin production while significantly increasing the yield of iturin [103]. Furthermore, the mutation of *fenC*, a fengycin biosythesis gene (Figure 1A), increased iturin production while mutating *ituC*, an iturin biosynthesis gene (Figure 1A), increased fengycin production [104]. By observing these discoveries, we can confirm that the pathways of biosurfactant production share a strong correlation with each other, implying the use of similar precursors and regulators, while specific regulatory mechanisms remain to be explored [92].

## 4. Biosurfactant Toxicity

Most chemically synthesized surfactants widely available on the market resist biodegradation and accumulate in nature. On the other hand, Biosurfactants are, like all-natural products, susceptible to degradation in water and soil [14]. Other than that, they are known for being less toxic or even non-toxic to the environment when compared to their synthetic counterparts and, thus, being the better choice when degrading pollutants. Some studies were already published comparing synthetic and natural surfactants and show how biosurfactants can be less toxic or even non-toxic at all [105,106]. However, Edwards et al. (2003) determined that the difference may not be significant [107]. Still, there are issues regarding the conditions in nature and the concentration and availability of biosurfactants in said situations, as they can vary between species and compounds, and lab conditions cannot properly mimic that.

Another concerning issue about biosurfactants is that some of the most significant producing organisms discovered so far are human pathogens and, thus, offer some degree of risk in their cultivation [108,109]. For example, one of the best-known bacteria capable of producing rhamnolipids is *P. aeruginosa*, a pathogenic organism [63]. Other examples of pathogenic species include the lipopeptide producers *S. marcescens* and *Inquilinus limosus* KB3 [110,111]. Hence, research on viable alternatives is steadily increasing over the years. According to Marchant et al. (2014), special attention to molecular biology techniques and quality bioinformatics assessment should be encouraged when studying alternative biosurfactant producers, as many studies being published are not replicable or are offering erroneous phylogeny; creating hindrances to getting better results and finding alternatives to pathogenic organisms [63].

## 5. Emerging Strategies for Biosurfactant Production

Being able to implement the newly discovered compounds is needed. As mentioned before, biosurfactants still struggle to compete with synthetic surfactants on large-scale applications globally. With that in mind, recent strategies have been developed to turn biosurfactants into competitive alternatives for industrial applications.

One such application is solid-state fermentation (SSF). A strategy for biosurfactant production that is geared towards overcoming the foaming encountered in the more popular submerged fermentation (SmF), this is used not only for biosurfactants but also other bioproducts [112,113,114]. This methodology has already been applied to the peptidic biosurfactant surfactin,. By using a medium based on okara, with the addition of sugarcane bagasse as a bulking agent, SSF was employed for surfactin production by *B. pumilus* UFPEDA 448 [115]. Other examples include SSF of soybean flour and rice straw as a substrate for the production of an antimicrobial lipopeptide by *B. amyloliquefaciens* XZ-173 [116], lipopeptide production by *B. subtilis* SPB1 grown on a mixture of olive leaf residue flour and olive cake flour [117] and surfactin production via SSF with rapeseed cake mixed with bacterial solution [118].

When producing biosurfactants, one of the critical elements is the media constituents. They play a role in the type and quantity of biosurfactant produced [3]. A technique that exploits this is using a carrier in the growth medium, which proved to be efficient in some cases, although not much has been done in this field yet [119,120]. When evaluating the lipoprotein biosurfactant produced by *Pediococcus dextrinicus* SHU1593, a study showed that using molasses and date syrup improved biomass formation [121]. Another study on *B. subtilis* CN2 showed that carbon sources on a hydrophobic substrate yielded much better activity and quality of the biosurfactant compared to a hydrophilic substrate [122]. 

The market always favors lucrative processes, and with that in mind, another feasible option for biosurfactant production is the co-production with other economically necessary products in a single bioprocess. Microorganisms tend to synthesize biosurfactants and other compounds, which could be taken advantage of. Before, co-production of pectinase and biosurfactant was observed in a *B. subtilis* strain isolated from a fruit dump yard [123].

As commented in a previous section, there are many pathogenic biosurfactant producers. To circumvent that, another alternative that tries to solve both the large-scale production and pathogenicity problems is using non-pathogenic microorganisms via recombinant production. For example, the gram-negative bacteria *S. marcescens*, an opportunistic human pathogen, is capable of producing serrawettin W1, a lipopeptide biosurfactant, and the NRPS protein SwrW is what catalyzes it [124]. By amplifying this protein, a recombinant production was established using the model organism *Escherichia coli* and a 20-fold increase in serrawettin W1 yield were achieved when compared to *S. marcescens* [108]. Another successful application of heterologous expression in *E. coli* occurs when using it as a vector for the hydrophobin protein DewA, which is naturally produced by the fungus *Aspergillus nidulans*, as it efficiently secretes it and provides facilitated purification [80]. There are still some hardships when approaching this method; for example, the recombinant expression of class I hydrophobin biosurfactant HGFI in *Pichia pastoris* has a somewhat lower yield after purification [83,125]. Also, when some hydrophobins are expressed in the model bacteria *E. coli*, the vector fails to deliver as it produces the biosurfactant in inclusion bodies or is often unfolded/misfolded [83].

## 6. Physical and Chemical Properties of Biosurfactants

**Figure 2:**
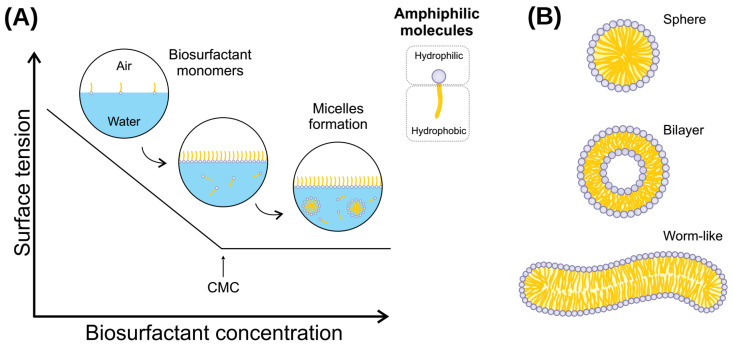
(**A**) Relationship between biosurfactant concentration and surface tension. CMC is defined by the concentration at which biosurfactant monomers form micelles and can reduce interfacial tension. (**B**) Different types of biosurfactant aggregates. A spherical shape occurs when the solution reaches CMC, and may become asymmetrical (such as a worm-like shape) at higher biosurfactant concentrations. Likewise, temperature or other environmental conditions induces the transition from spherical shape micelles to micellar vesicles in biosurfactant assemblies.

Biosurfactants are bioderived or biomimetic surfactants that can act as detergents, wetting agents, emulsifiers, dispersants, and foaming agents [126,127]. These molecules have the properties of reducing water surface tension and decreasing water/oil interface tension (Figure 2) [3]. This occurs because these amphiphilic molecules can dispose of the limit of surface water and assemble into micelles, which can shield hydrophobic molecules present in the solution from the unfavorable interactions with water molecules [65]. Moreover, biosurfactants present adsorptive properties on surfaces and interfaces [128].

Biosurfactants are molecules that present a wide range of industrial applications, such as cleaning (laundry products), biofilm prevention and disruption, biocidal activity, wound healing, and various uses in the petroleum industry and oil bioremediation [127,129]. This array of applications comes from the particular chemical features that biosurfactant molecules present. From a physicochemical perspective, (bio)surfactants are amphipathic molecules that lower surface and interfacial tensions. Consequently, water-immiscible substances will increase their solubility at the surfactant-water interface [3]. The molecules will spontaneously aggregate into micelles under non-extreme conditions and at a minimum concentration. For this reason, understanding the micelle formation process is of pivotal importance for the application of biosurfactants. The emulsifying ability of each biosurfactant is the result of the micelle characteristics it will form, and the micelle characteristics are the consequence of the isolated molecule properties [130].

The CMC and the aggregation number are the two main parameters to investigate in a new biosurfactant. When biosurfactant molecules are slowly added to water, the molecules will stay only on the surface (at a very low concentration). At this point, there are no micelles in the solution. With the slow increase of molecules in the solution, it will reach a concentration in which no micelles are yet stably assembled at a certain point. Still, more biosurfactant molecules will promote the formation of thermodynamically stable micelles. This specific concentration is called CMC (Figure 2). The number of molecules that constitute a micelle formed just as CMC has been overreached is defined as the aggregation number [131]. There are different methods to determine both the CMC and aggregation number of a given biosurfactant, such as isothermal titration calorimetry [132], THP-based determination method [133], fluorimetry, conductometry, and surface tension [134]. Furthermore, CMC depends on temperature, pressure, pH, and ionic strength; thus, investigating the relationship between CMC and those parameters is utterly essential.

As stated before, CMC revolves around the concentration where micelles will assemble spontaneously. Thus, standard Gibbs free energy of micellization (δGom) is calculated relatively to CMC, as shown in Table 2. Note that ionic micelles are more sensitive to salt concentration, thus taking into account free counter-ion concentration. Moreover, the standard enthalpy of micellization (δHom) indicates if the process is endothermic or exothermic for a given biosurfactant. Many biosurfactants, such as peptide-derived ones, are ionic or zwitterionic molecules, which in most cases present an endothermic micellization process, where the negative free energy is a compensation in the system’s entropy increase (δSo = δSom + δSosol) [135]. This observation implies that the micellization process is driven by the water molecules surrounding the biosurfactant rather than a strong interaction between the biosurfactant monomers. Thus, organisms that are dependent on this type of biosurfactant to survive should be adapted to environments where the ionic strength will not be enough to organize and restrain water molecules in such a way that will lower the solvation entropy or possess a secondary mechanism that will compensate or reduce the ionic strength. It is also important to note that when considering the effect of ions, it can not be assumed a monotonic behavior for the micellization process [136].

An essential aspect of biosurfactants CMC and its aggregation number is how temperature affects the assembly process. Attempting to predict CMC as a function of temperature is not a straightforward process. That is because surfactants, in a general perspective, vary considerably in their intrinsic structural features, such as ionic and non-ionic nature, hydrophobic moiety size, polar moiety size, and the number of hydrogen bond possibilities. Whereas some studies predicted a monotonic behavior between CMC and temperature for different surfactants [137,138], new evidence suggests that the effect of the temperature on the CMC is non-monotonic and the monotonic behavior is observed only in a specific range of temperature [139] (see Table 2 for the equations). This observation is due to the hydrophobic effect between the surfactant tails and the polar solvent molecules. The transfer of the surfactant n-alkyl tail from the aqueous solution to the hydrocarbon core of the micelle is the main energetic contribution to the micellization process. This way, an increase in the length of the n-alkyl tail should increase the hydrophobic effect and lower the CMC, but other variables, such as tail deformation, should be considered. Therefore, we observe a non-monotonic relationship between n-alkyl chain length and CMC [140].

Moreover, a study about the effects of temperature on micelle formation in apolar media showed that SPANs, which are sorbitan oleates, will form reverse micelles. Increasing the temperature will increase the critical micelle concentration of most surfactants, suggesting that the primary state function driving reverse micelle formation in apolar solvents is enthalpy. Only one surfactant showed an entropically driven micellization process, where increasing the temperature resulted in a decrease in CMC [141]. This sort of investigation is most pertinent, primarily when intended to employ biosurfactants in the petroleum industry or for bioremediation of oil spilled areas since petroleum is an admixture mostly of hydrophobic compounds.

Other important physicochemical parameters of biosurfactants that should be looked at more in-depth are surface and interfacial tensions [34]. As mentioned before, solvents present a cohesion related to their intermolecular interactions, which results in the solvent’s propensity to resist rupture when exposed to an external force. In this sense, biosurfactants’ properties can lower this cohesive force, increasing the solubilization of poorly soluble substances. The lowest surface/interfacial tension is achieved when the biosurfactant concentration reaches CMC [142]. Thus, surface and interfacial tensions are related to CMC. The lower the concentration required for a given biosurfactant to assemble micelles is the concentration where surface tension is minimum. More practically, when adding biosurfactant molecules to a pure water system, the solution’s surface contains a much higher concentration of biosurfactant molecules than the bulk, even when CMC is reached. Since the water surface spontaneously presents a higher biosurfactant concentration than the bulk, increasing the water surface area would result in a withdrawal of biosurfactant molecules from the bulk to compensate for the new surface. In this scenario, if the biosurfactant concentration is increased, the bulk will present a higher concentration, increasing the chemical potential [143]. Consequently, it will be easier to withdraw molecules from the bulk when the surface area increases, lowering the surface tension.

One concept that comes to be important is the distinction between biosurfactant and bioemulsifier compounds. Even though they present similar structural features and some chemical properties, they have enough remarks to categorize them, as reviewed by Uzoigwe et al. [36]. The first difference between these two molecule classes is their size. Bioemulsifiers are higher in molecular weight compared to biosurfactants. Chemically, they are biopolymers or exopolysaccharides, constituted of a complex mixture of heteropolysaccharides, lipopolysaccharides, lipoproteins, and proteins.

On the other hand, as mentioned before, biosurfactants are oligosaccharides, amino acids, and fatty acids derived compounds, presenting a considerably lower molecular weight than bioemulsifiers [36]. Another difference regards the ability to lower the surface and interfacial tensions. Whereas both compounds can efficiently emulsify two immiscible liquids or poorly soluble compounds, biosurfactants are more effective at reducing the surface tension [144]. The chemical aspect of these classes of compounds might rest some clues about the evolutionary traits of organisms adapted to produce them. To further discuss the biological implications, first, we must take an additional glimpse into the thermodynamics of surface tension, especially its relationship with temperature and concentration, along with the biosurfactant solubility nature.

As for the CMC, surface tension is also dependent on temperature. Generally, interfacial and surface tensions decrease with temperature increase [145]. This observation is due to a system’s energy compensation. When molecules are transferred from the bulk to the surface, there is an increase in the system’s energy. That is due to the balance between intermolecular attractive and repulsive forces of neighboring molecules and the energy necessary to overcome those forces for motion. Given that this is a spontaneous process, the gain of energy is compensated by reducing the surface area and, consequently, surface tension. At higher temperatures, the greater kinetic energy of the molecules results in a decrease of the attractive forces, exacerbating the reduction of surface tension. Experimentally, the relationship between temperature and surface tension was modeled by different scientists throughout history. Eötvös found a correlation between temperature and volume-based surface area, and Guggenheim found a formula based on the property of each liquid [145,146], observing the same temperature-surface tension relationship.

### 6.1. Surfactin and Hydrophobin—Case Studies

Surfactin is one of the most well-studied biosurfactants, and surfactin-like molecules have recently been extensively reviewed by Théatre et al. [147]. Its structure is a cyclic lipoheptapeptide composed of L-Glu1-L-Leu2-D-Leu3-L-Val4-L-Asp5-D-Leu6-L-Leu7, which assembles into a lactone ring structure with a β-hydroxy fatty acid chain [148,149]. Multimodular mega-enzymes synthesize the peptide moiety denominated non-ribosomal peptide synthetases [43]. On the other hand, the fatty chain moiety is synthesized through the classical fatty acid biosynthesis pathway [150], followed by the production of 3-hydroxy-acyl-coenzyme A [151]. Later, this substrate is connected to the peptide moiety through the action of the surfactin synthetase [152]. Several structural variations are observed in surfactin molecules produced by different *Bacillus* species. Amidst the diversity of structural features documented, there are Val4Leu, and Leu7Ile/Val (pumilacidin) [153]; Glu1Gln (lichenysin) [154]; fatty acid chains varying from 12 to 17 carbons, whereas the most common length is 14-15 carbons [147,155]; isomers in the lipid chain, where it can be linear or branched, iso and anteiso; linearization of the peptide moiety (not frequent) [156]. Additionally, a modified C15-surfactin-O-methyl was seen in *B. subtilis* HSO121 [157] and C14-surfactin methyl ester in *Bacillus pumilus* KMM 456 [158]. There are also synthetic structural modifications in surfactin that alter its natural properties, such as esterification [159], linearization of the cyclic surfactin [160], epimerization of L-Leu2 to D-Leu2, charge modification when Asp5Gln, and the switch of residues Asp4-Leu5 [161].

As discussed before, there are various physicochemical properties of biosurfactants, and their understanding is pivotal for industrial or bioremediation applications and for understanding the biosurfactant-producing organisms’ evolutionary process. For surfactin, the CMC for a C15 fatty acid moiety is 20 µM in Tris-HCl pH 8, and the CMC increases as the hydrophobic chain length decrease [162]. This is a result of the packing of surfactin molecules during the micellization. Longer fatty acid chains allow an improved hydrophobic core assembly once the peptide ring is large and hinders the linear chain contacts. Therefore, branched chains are expected to decrease CMC since it is unfavorable to van der Waals interactions. Moreover, linearization of the peptide moiety increases the CMC significantly [163]. Another aspect, the micelle geometry can vary considerably according to the system’s parameters, such as temperature, pH, ionic strength, and surfactin concentration [164,165]. In this sense, surfactin micelles’ shape can be sphere-like, worm-like, and unilamellar bilayers [1].

Another property of biosurfactants previously discussed is surface tension. Surfactin reduces surface tension when in CMC and Tris pH 9.4 to 37 mN/m. The lichensyin variant reaches 35 mN/m under the same conditions [166]. Additionally, lichensyin CMC is tenfold lower than surfactin in the absence of cations [166]. The fatty acid chain influences the surface tension reduction of surfactin, whereas the linear, long-chain is more efficient.

Another type of biosurfactant is hydrophobin, which has also been reviewed over the last years [42,167,168]. Hydrophobins are small, surface-active proteins produced by filamentous fungi. This protein is structurally characterized by a β-sheet core with four anti-parallel β-strands, containing eight conserved cysteine residues, which form four disulfide bridges, and an external α-helix. Hydrophobins have been proven helpful in various industrial applications, such as protein purification processes, biosensors, drug solubility, food dispersion, and tissue engineering. Furthermore, hydrophobins are classified into two groups based on their solubility: class I and II.

Molecular dynamics elucidate hydrophobins’ molecular behavior and physical-chemical properties. Euston [169] investigated the adsorption property of the class II hydrophobin HFBI at different interfaces: vacuum-water, di-palmitoyl-phosphatidylcholine (DPPC)-water, and decane-water. HFBI showed, generally, an anisotropic structure characteristic, acting mainly as a rigid body when adsorbed and presenting high structural stability. However, the protein’s behavior is dependent on the type of interface. The biosurfactant displays spontaneous dimerization conduct in water, aggregating by their hydrophobic patch. On the other hand, HFBI showed a propensity to denaturate its surface and lose secondary structure, reducing its β-structure when at the decane-water interface. Moreover, HFBI change in its tertiary structure is slightly more significant at the DPPC-water interface when compared to the vacuum-water interface.

Next, Raffaini et al. [170] investigated the molecular behavior of HFBII in water, fluorinated solution, and water-vacuum interface at an atom level. HFBII showed only minor local changes in the hydrophobic patch when in the fluorinated solution; As expected, when in water, HFBII relocates to the water-vacuum interface, keeping the hydrophilic residues in contact with water and exposing its hydrophobic side to the vacuum. These results are essential for future investigation of the HFBII self-assembly process.

### 6.2. Physical-Chemical Selective Pressure of Biosurfactant Molecules

What do the physical-chemical aspects of surfactants reveal about evolution of the biosurfactant-producing organisms? Temperature, pH, and ionic strength are the parameters that vary the most in different environments. Organisms from fresh, warm water are subjected to different evolutionary pressures than organisms from brine, cold water. Higher temperatures induce biosurfactants to quickly lower surface tension, while low-temperature environments are more challenging to reduce surface tension. Thus, from a physical-chemical perspective, it can be expected that organisms from cold environments present a more extended, less-branched hydrophobic chain to compensate for the temperature barrier for lowering surface tension.

**Figure 3:**
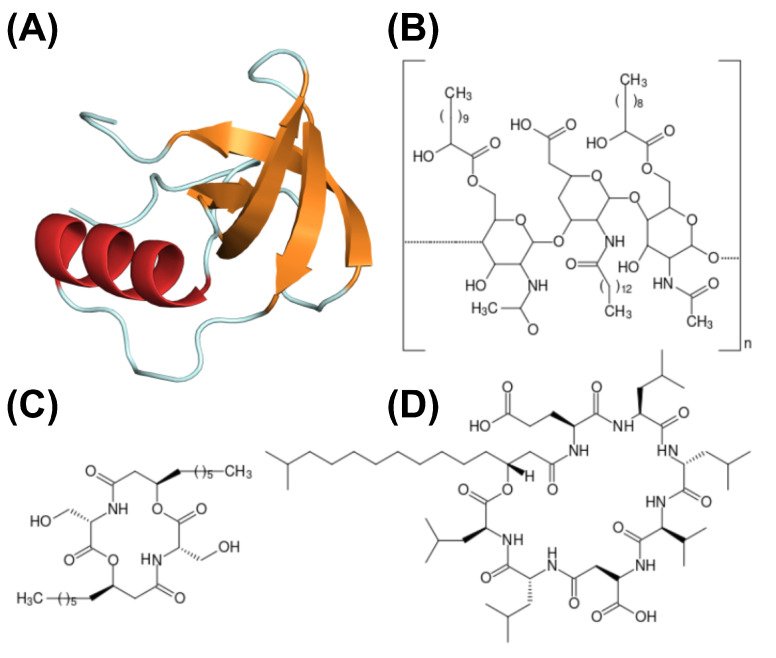

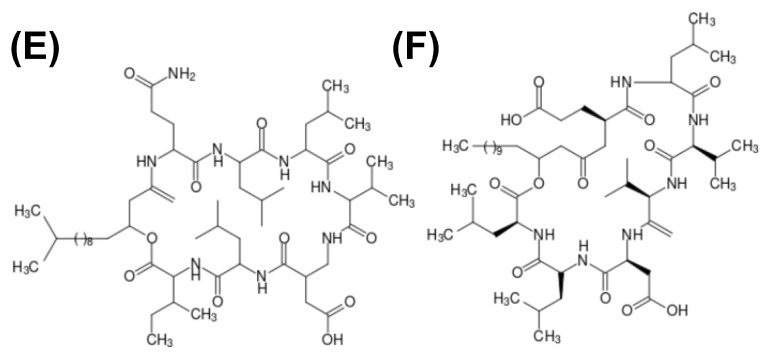
Structure schematics of biosurfactants. (**A**) Hydrophobin (PDB ID: 2PL6), structure of β-sheet core represented in orange, and external α-helix colored in red; (**B**) emulsan, a polymeric biosurfactant; (**C**) serrawerttin; (**D**) surfactin; (**E**) lichenysin; (**F**) iturin.

Biosurfactant properties are intrinsically connected to the molecule structure. Small proteins, like hydrophobin, will display a different molecular behavior compared to small, peptide-derived biosurfactants. Peptide and protein biosurfactants may present different net charges, molecular polarity distribution, alkyl chain length, molecular mass, and other characteristics that influence their biosurfactant properties. Moreover, biosurfactants may present as polymers, such as emulsan. Figure 3 depicts the structure of different biosurfactants.

## 7. Omics Technology and Bioinformatic Analysis as a Tool for Biosurfactant Identification

The appropriate use of biosurfactants, in general, is only possible when metabolic and bacterial diversity is known. Understanding how interaction occurs among microbial communities is essential to elucidate biosurfactant production. The richest source for biosurfactant-producing microorganisms is seawater, as the conditions of this environment allow for the survival of only the most tolerant organisms [171]. Nevertheless, even though around $1\times 10^{6}$ bacteria are present in 1 mL of seawater, only about 0.0001–0.1% of these are culturable [171,172]. The same goes for soil microorganisms, where less than 1% of those are cultivable [173].

**Figure 4:**
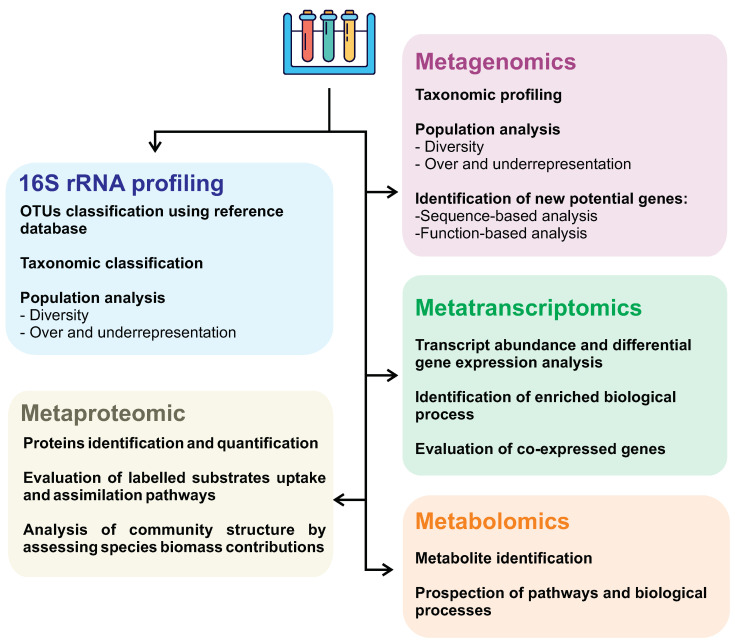
Omics approaches and their applications as strategies in biosurfactant research.

First described in 1998 as the total of genomes found in a sample [174], metagenomics is the analysis of genomes contained within an environmental sample with no need for prior cultivation or classification of the species [175]. Therefore, this opens a wide array of opportunities to discover novel species and compounds, as said before, because most of the microorganisms are uncultivable in lab conditions. Not only that, but the natural conditions *in situ* that these life forms exist are so complex that reproducing them artificially is practically impossible. On top of that, metagenomic and genetic data, in general, are widely available for use in public databases over the internet, making the information much more accessible for use. In addition, other large-scale approaches can also be employed, as shown in Figure 4.

Metagenomics allows us to access the genetic information of microorganisms that cannot be cultured in laboratory conditions, allowing environmental DNA extraction and the evaluation of microbial consortium, which can significantly contribute to the discovery of new biosurfactant producers and molecules. In contrast, 16S rRNA gene sequencing can’t retrieve the microorganisms’ genome being able to give an accurate microbial composition profiling sampled environment. Metagenomic studies consist of main approaches: (i) sequence-based, and (ii) function-based strategies. Sequence-based analyses have steadily advanced over the years alongside next-generation sequencing platforms. This technique focuses on discovering protein-coding sequences based on homology using curated data from databases. This method has been used to identify genes that regulate biosurfactant production in the genome of *S. marcescens* strain Db10 [171,176]. Despite its success, sequence-based screening is widely believed to be limited. It arises from the fact that existing homologous genes are being targeted, and very few novel genes are detected [171,177].

The function-based analysis involves screening the metagenomic library for functional activities from the expression of genes in the bacterial metagenomic DNA [178]. This approach is much more time-consuming and laborious than homology-based approaches but yields better results in identifying novel genes [177]. Even so, this technique is ever-evolving, and with time several different methods were elaborated and put into action to make it even more efficient [179].

The metagenomic approach has been growing for the past few years as an efficient way to elucidate biosurfactant production and bioremediation efficiency [180,181,182,183]. Some key biosurfactant producers identified in marine samples are the *Bacillus* and *Rhodococcus* genera [184]. Examples of novel biosurfactants discovered in saltwater environments are Aneurinifactin [185], a laccase-like enzyme [186], and an esterase called EstATII found in the red sea [187]. Although described in soil, the archaeal biosurfactant MBSP1 is thought to have great efficiency in marine environments [188].

Moreover, soil contamination has also been in the spotlight, as it is an environment considered of vital importance and one of earth’s “system life support” [189]. Metagenomics is a pivotal component in understanding soil contamination and has been used in soil-oriented biosurfactant research. One example is hydrocarbon-contaminated arctic soil in Canada, where the soil metagenome was sequenced over time. As results, *Pseudomonas* was the most active microorganism during peak contamination, followed by *Rhodococcus* [190]. Experiments followed over time allow monitoring the microbial population dynamics and its variation as a response to the interaction of organisms with each other and with the environment. Furthermore, evaluating multiple interacting organisms better reproduces the biological reality that will be faced in most biosurfactant applications.

Different omic approaches (such as metagenomic, metatranscriptomic, metabolomic, and metaproteomic) can evaluate different levels of knowledge of a system and, therefore, should be chosen and combined to address the research aim better. Despite metagenomics being more widely used in the investigation of new biosurfactants, the combination of multi-omic approaches contributes to greater accuracy in investigating surfactant biosynthesis pathways, providing a more detailed description of interacting microorganisms, especially in taxonomically very diverse communities. Metagenomic analyses give an insight into the present microorganisms, their abundance, and the functional aspects of their genome. On the other hand, metatranscriptomics can better understand the whole gene expression profile, shedding light on microbial communities’ ecological response subjected to specific environmental conditions, such as oil spills. In other words, metatranscriptome allows us to assess which molecules are active at a given time and how abundant they are, discriminating members actively contributing to a specific response or behavior from those quiescent members.

Other omics approaches, such as metabolomics and metaproteomic, are barely discussed in the literature searching for new biosurfactants. One possible reason is the high cost of these analyses associated with numerous challenges related to the analysis workflow. Metaproteomic generally shows lower measurements depth than metagenomic and metatranscriptomic, and protein inference may be challenging due to redundant identification caused by homologous proteins [191,192]. However, with a highly curated and comprehensive species protein database, it is possible to precisely identify and assign these proteins to their respective taxa, providing a picture of microbial activity and the exploration of novel functional genes and biochemical pathways in response to specific conditions [193]. Despite this, proteomics can generate exciting results, as demonstrated by an analysis conducted by Pitocchi et al. from two fungi species isolated from the spill-oil polluted marine site [194]. In this research, it was identified a potent biosurfactant secreted by *Aspergillus terreus* MUT 271 and *Trichoderma harzianum* MUT 290 belonging to cerato-platanins protein family with similar properties of hydrophobins [194].

Metabolomics provides a global picture of metabolites, reflecting the biochemical activity of microorganisms. In microbiomes analysis, it is challenging to relate metabolites to specific taxa; being possible to solve this issue by identification of covariates metabolite integrated with a species composition analysis, which may indicate species-specific metabolite production [191]. Interestingly, an untargeted metabolomics study from one thousand marine isolates microorganisms identified several molecule classes and annotated 76 molecular families, among them, the surfactin biosurfactant [195].

Studies at the biological system level open the opportunity to explore new molecules and identify new organisms that produce biosurfactants, generating knowledge about the ecological systems where these organisms are found. Knowledge bases on biological pathways and functional classification are constantly expanding. A specific repository to assist in biosurfactant research has already been launched, BioSurfDB, which contains information on metagenomes, organisms, cured biosurfactants, bioremediation experiments results, and biodegradation relevant genes Oliveira et al. [196].

Collecting samples and sequencing are only some essential steps in omics studies. After gathering data, all that information needs to be analyzed through bioinformatics methods. In addition to the well-established workflows appropriate for different omic data, the integration of omic analyzes could lead to more consistent and informative results.

### 7.1. Network Analysis

Understanding the relationship between molecules and the organization of possible molecular pathways associated with biosurfactant production is crucial. Interaction networks are valuable tools to deal with a massive amount of information, whether from a single methodological approach, such as protein-protein interaction networks or a combination of data from multiple levels of knowledge. These networks represent direct and indirect interactions between the components of that system, which can describe genes, proteins, and any other target molecule. It is possible to build networks from multiple omics approaches, linking the results of genomics, transcriptomics, proteomics, and metabolomics within a single system through multilayer networks. Numerous works have used interaction networks to understand the relationship between genes/proteins and different compounds [195,197,198,199]. Also, metabolomic-based networks were used to elucidate metabolic profiles and chemical structures from secondary metabolites produced by *S. marcescens* strains, resulting in the identification of serrawettin W1 [200].

However, the integration of multi-omic data is a challenge on its own. Compiling different data layers requires high computational costs and robust pipelines that ensure the quality of the combined information. Additionally, creating consistent workflows requires substantial knowledge of the analyzed systems. Identifying modules capable of participating in a common biological function during network analysis is essential for understanding a complex network’s structural and functional aspects. Many modules are conserved throughout evolution and may represent core functions or processes for biological regulation [201]. Clustering and topological analysis are powerful methods for elucidating molecular machinery. It is not unusual in omics analyses to identify new molecules of unknown function. Although sequence-based analysis is usually used, module analysis can be very useful for applying a principle called guilt-by-association, especially in co-expression networks [202]. In this sense, poorly annotated genes within the same module are more likely to be associated with similar processes. However, this approach must be undertaken rigorously to avoid misinterpreting these potential genes [202].

Significant advances also have been made in the area of flux balance analysis (FBA). This research area estimates metabolic flux in genome-scale metabolic network reconstructions using the constraints imposed by stoichiometric coefficients of each reaction [203]. FBA can be used for a wide variety of applications, being able to incorporate data from different omics. Thus, it is possible to create FBA models to study the tolerance of an organism to mutations and physiological stress, the organism’s growth rate, or even the production rate of a specific metabolite. FBA analysis can be of great importance for metabolic engineering, whose knowledge can provide insight into the large-scale production of biosurfactants. Occhipinti et al. [204] used in silico engineering to introduce the RhlA and RhlB genes and reactions responsible for rhamnolipids biosynthesis from *Pseudomonas aeruginosa* to an existing genome-scale model of *P. putida*. In silico models can be useful to predict the metabolic and genetic engineering steps needed for maximizing the production of a target compound.

Systems biology becomes a great ally in understanding microbial diversity and composition and in analyses related to cellular processes and responses to certain conditions. Furthermore, understanding natural or endemic bacterial communities in regions that require attention, such as crude oil spill-affected regions, can aid prospecting for biosurfactant-producing microorganisms. Different network analysis methods for studying microbial communities have been proposed and are explored in detail in [205,206]. A study conducted in a microcosm showed that microbial biofilm diversity decreased after exposition to the biosurfactant rhamnolipid. Furthermore, exposure of the microbial community to rhamnolipid considerably changed extracellular enzyme activity, reducing phosphatase activity and increasing beta-glucosidase levels. Network analysis can also be applied to assess the impact of surfactant additions on natural microbial communities to support (or not) their applications once their impact on the environment is proven. In this sense, a study in a northeast Atlantic marine community exposed to crude oil spills investigated how the biosurfactant rhamnolipids or synthetic dispersants could impact community dynamics and functional diversity. The synthetic surfactant negatively affected taxa diversity and impacted the microbial community’s functional robustness more significantly than biosurfactant use.

### 7.2. Machine Learning and Data Integration

Machine Learning (ML) is a field derived from studies of artificial intelligence, pattern recognition, statistics, and optimization, which aims to develop algorithms capable of performing functions without being explicitly controlled by a user. ML techniques “learn” how to make predictions and decisions through a given data or by integrating more than one [207,208], which could be genomic, transcriptomic, proteomic, or metabolomic. The main advantage of using ML is its ability to extract information from a massive amount of large-scale data, which can be used to create predictive models for a given situation quickly and with high precision, in addition to finding essential patterns for the description of the biological phenomenon [209].

Analyzing the omics datasets in an integrative way using ML techniques will return a complete picture of the whole system than separately analyzing them. Data integration can be defined as the integrative study of data from multiple sources to improve knowledge discovery [210,211,212]. Tarazona et al. [213] categorizes integration methods based on incorporating additional biological and using supervised or unsupervised approaches. Supervised methods can be used in two principal ways—the first is for predicting a response variable. The second way is to understand the communication between the omics layers or, more specifically, to model the potential regulations to build the regulatory network of the biological system studied [214]. Unsupervised methods are mainly applied for a preliminary exploration of datasets and sometimes also for clustering observations. In the case of multi-omic experiments, an unsupervised strategy can help us to understand the relationship among omics and find common and different patterns among them. Unsupervised strategies comprise clustering, dimension reduction methods (e.g., principal component analysis, T-distributed stochastic neighbor embedding), or machine-learning strategies (e.g., artificial neural networks).

Machine learning approaches have been increasingly applied to predict suitable parameters to improve biosurfactant production yield for scale-up toward industrial production. Bioprocess engineering involving biosurfactant production faces several challenges since different factors affect its production, such as carbon and nitrogen sources, temperature, pH, oxygen requirements, metal ions, and agitation speed [215]. The incorrect adjustment of these factors could affect the system’s efficiency, influencing the microorganisms’ growth and metabolic activity [215]. In this sense, different machine learning algorithms have been applied to optimize biosurfactant production, such as artificial neural network (ANN) [216,217], support vector regression (SVR) and support vector regression coupled with firefly algorithm (SVR-FFA) [218], and support vector machine (SVM) [219].

Interspecific interactions within a population are paramount to understanding the relationships in a synthetic consortium or between the microbial community and their environment. In this sense, Chang et al. applied ML using the Random Forest method on metagenome data to assess whether the composition of microorganisms is related to crop productivity [220]. Interestingly, using ML approaches, it was possible to identify a subset of taxa that could be responsible for functional aspects of the community [221]. Also, a study showed that support vector regression-fruit fly optimization algorithm (SVR-FOA) was able to accurately estimate the simultaneous biodegradation of azo dyes and hexavalent chromium by a biosurfactant-producing bacterial strain *Klebsiella* sp. KOD36 [222]. ML applications in microbial analysis focusing on data integration and understanding cooperative and competitive relationships within a microbial ecosystem are an important direction for future research.

### 7.3. Molecular Dynamics (MD) Simulations

The principle behind MD is to represent computationally molecules and ions, which can be at an atomistic level or coarse-grained; define a volume for the experiment to take place, which will be represented as a box, and apply Newtonian physics principles in the system to calculate all bonded and non-bonded forces in motion. In other words, MD allows us to use computer processing to simulate and visualize how a set of molecules behave under specific conditions in a given time. From one typical simulation, one can obtain a myriad of information about structural features and physical-chemical properties. Moreover, a molecule, or thousands of the same molecule, such as a specific biosurfactant, can be simulated in different solvents. This way, the properties in different interfaces can be investigated [223,224,225].

Furthermore, MD can be performed at varying temperature, pressure, ionic force, solvent type, the number of particles (concentration), and other parameters that might be relevant to a specific experiment. Therefore, one can investigate structural features, such as hydrogen bonds through time, atoms position and fluctuation through time, as well as physical-chemical properties like density and free energy of solvation. A handful of free software programs for MD, such as GROMACS [226], NAMD [227] and OpenMM [228]. An easy, straightforward tutorial to learn how to simulate from scratch using GROMACS can be found in [229].

Moreover, surfactants properties, such as CMC, interfacial/surface tension, Winsor I-II-III-IV transition, Hydrophile-Lipophile Balance (HLB), and structure-property relationships can all be assessed through MD techniques [230]. Furthermore, variations of MD have been developed to investigate free energy-based processes to overcome limitations from classical, atomistic MD simulations. Since MD keeps

Assessing CMC through computational methods depends on the calculation of the aggregation probability number (P(N)), which is the likelihood of N molecules aggregating [230]. However, the minimum of biosurfactants molecules needed to produce a micelle varies depending on the system. In this sense, MD can predict biosurfactants’ aggregation number, structure, and CMC. It has been employed before to study a variety of surfactants with different properties, such as ionic [231], and nonionic [232].

Next, MD simulations have been employed to investigate interfacial/surface tension, which is accomplished based on mechanical laws through pressure tensor calculation, or the system’s free energy derivative concerning the surface area [230]. For surfactants in general, experiments at the wet lab and MD simulation results correspond well, especially regarding the density distribution of alkyl chains and surfactant headgroups. However, MD cannot forecast the counterion distribution effectively, leading to the opposite results in experiments and modeling for the counterion affinity with the surfactant headgroup [233,234,235]. The core reason for this limitation is that by utilizing default Lennard-Jones values from classical force fields, the attraction between the surfactant headgroup and the counterions may be exaggerated. By lessening this attraction, it is possible to achieve a surfactant/counterion interaction that is realistic and consistent with the experimental findings [236].

Furthermore, it is possible to employ MD to investigate Winsor transitions [230], which are related to the different types of microemulsions. Four different types of microemulsions, known as Winsor-type microemulsions, exist [237]. Type I microemulsions are defined by the solubilization of oil in spherical, normal micelles within the water-continuous phase; type II presents solubilization in reverse micelles within the oil-continuous phase; type III exists in three-phase systems in which the middle phase microemulsions remain in equilibrium with both aqueous and organic phases and; type IV, characterized when the middle phase microemulsions are expanded at a high surfactant concentration, resulting in the formation of a single phase from the combination of aqueous and organic phases [238]. Moreover, parameters such as the temperature, the surfactant concentration, and the salinity all affect how many phases a system consists of (bio)surfactants, an organic phase, and a brine, aqueous phase. Changing the parameters will result in a phase transition. Thus, performing simulations with systems at different temperatures, types of biosurfactant (i.e., different size, shape, and hydrophile/hydrophobic moieties proportion), and nature of the organic phase will influence Winsor I-II-III-IV transitions.

Another important property of amphiphilic biosurfactants, such as lipopeptides, is the HLB. A biosurfactant’s HLB is the balance between the size and the tensile strength of its hydrophilic and lipophilic moieties [239]. Among the structural features that influence lipopeptides self-assembly, HLB is one important parameter to consider. Studies have indicated that the minimum lipid moiety size is crucial for self-assembly. However, there is no universal rule to predict such property since the sequence and length of the peptide moiety also influence amphiphilicity. Many other factors, including electrostatic interactions, hydrogen bonding, van der Waals interactions π-π stacking interactions [240]. In this sense, MD simulations provide an in-depth analysis of all these factors, and investigation of systems varying temperature, molecule type, and salt concentration is easily achieved. Additionally, non-bonded interactions are routinely discrete by MD analyses, thoroughly scrutinizing how much each factor contributes to the overall net result.

Structural analyses of biomolecules are, perhaps, the most common application of MD simulations. Understanding the molecular behavior of biosurfactants through time, such as the influence of specific intermolecular interactions, the impact of angle and dihedral conformations, the variation of position and fluctuation of atoms, solvent accessible area, and energy profile, are typical MD analyses. Moreover, these analyses are conveniently applied to evaluate the effect of biosurfactants on other proteins [241]. This approach has been employed for decades [223], and it is not only pertinent nowadays, but its use is compelling since computational resources become better over time and force field parameters are more accurate. A myriad of studies regarding MD simulations and peptide biosurfactants are available in the literature [223,225,241,242,243,244,245,246,247,248,249,250,251,252,253,254,255]. Moreover, specific reviews about MD and biosurfactants are available [256,257,258].

Although MD simulations have been consistently employed to investigate surfactants’ properties, only a few types of peptide biosurfactants were regularly assessed through the lens of MD. This way, there is a sea of opportunities for different works for MD and biosurfactants. Furthermore, MD simulations present as the nexus between physical-chemical theory and wet lab experiments. Biosurfactant studies employing MD techniques have been carried out consistently for a long time, and the results have shown to agree with the literature. Thus, incorporating computational methods in the experimental workflow will provide in-depth, comprehensive results to conduct global research at lower costs.

## 8. Conclusions and Perspectives

Lipopeptides are a class of compounds with a large diversity of structures and functions, resulting in a variety of applications as ecological roles. And, as we can see, the study of biosurfactants has been exponentially growing during the past years. While, as a subject of study, they are rather old, new research tools have enabled a much better understanding of biosurfactant production, composition, and function. More efficient ways of cultivating microorganisms and boosting biosurfactant yields and the combined production alongside other compounds of interest during co-production are here to ease the market for their use. While research using bioinformatics tools is giving us new ways to understand biosurfactants and discover new natural surfactants, as well as insight into how microbial populations function during contamination and how the composition of these populations can affect the biosurfactant yield. All the information and research tools discussed here are essential going forward on biosurfactant research. Although it seems like much is known in this subject, around 30% of bacterial lipoproteins are still not functionally characterized. And this is were powerful means of complex data analysis in molecular dynamics and meta-mics research come into play.

By using the technologial assets presented here, thorough studies on biosurfactants can be envisioned. For example, in a contaminated site the whole genomic and transcriptomic profiles of microbial populations can be sampled, analyzed, and these results compared in multi-field research. We can detect biosurfactant producers via metagenomics and metatranscriptomics, test biosurfactant yield and degradation capabilities in lab trials, and elucidate the physical and chemical properties of biosurfactant molecules, while understanding the environmental influence on their molecular behavior. Furthermore, machine learning methodologies can be used to predict output parameters of biosurfactants to optimize the conceptual phase of studies. Predicting the total biosurfactant production is also a great asset to understand total costs of practical applications and use of more cost-effective substrates.

Biosurfactant-producing microorganisms, especially from extreme environments, show great promise for the discovery of novel lipopeptides. As more powerful research tools emerge, such as metagenomics, metatranscriptomics, MD simulations and machine learning are coupled with traditional laboratory research methods, the discovery of diverse lipopetides structures and functions becomes possible.

## Figures and Tables

**Table 2:** Equations relating to the physicochemical processes involved with biosurfactants.

Name	Formula	Description
Micellization (non-ionic)	${\Delta G}_{m}^{0}=RT\ln X_{CMC}$	${\Delta G}_{m}^{0}$ = standard *Gibbs free energy* of micellization *R* = gas constant (${\mathrm{mol}}^{-1}K^{-1}$) *T* = temperature (K) $X_{CMC}$ = CMC in mole fraction
Micellization (ionic)	${\Delta G}_{m}^{0}=\left(2-\alpha \right)RT\ln X_{CMC}$	${\Delta G}_{m}^{0}$ = standard *Gibbs free energy* of micellization α = degree of dissociation of ioinic surfactants *R* = gas constant (${\mathrm{mol}}^{-1}K^{-1}$) *T* = temperature (K); $X_{CMC}$ = CMC in mole fraction
Standard enthalpy of micellization (non-ionic)	${\Delta H}_{m}^{0}=-2.303RT^{2}[\partial \log X_{CMC}/\partial T{]}_{P}$	${\Delta H}_{m}^{0}$ = standard enthalpy of micellization *R* = gas constant (${\mathrm{mol}}^{-1}K^{-1}$) *T* = temperature (K) $X_{CMC}$ = CMC in mole fraction
Standard enthalpy of micellization (ionic)	${\Delta H}_{m}^{0}=-2.303\left(2-\alpha \right)RT^{2}[\partial logX_{CMC}/\partial T{]}_{P}$	${\Delta H}_{m}^{0}$ = standard enthalpy of micellization α = degree of dissociation of ioinic surfactants *R* = gas constant (${\mathrm{mol}}^{-1}K^{-1}$) *T* = temperature (K) $X_{CMC}$ = CMC in mole fraction
Standard entropy of micellization	${\Delta S}_{m}^{0}=[{\Delta H}_{m}^{0}–{\Delta G}_{m}^{0}]/T$	${\Delta S}_{m}^{0}$ = entropy of micellization ${\Delta H}_{m}^{0}$ = enthalpy of micellization ${\Delta G}_{m}^{0}$ = *Gibbs free energy* of micellization *T* = temperature (K)
Entropy of micellization	${\Delta H}_{m}^{0}=\partial \left({\Delta G}_{m}/T\right)/\partial \left(1/T\right)$	${\Delta H}_{m}^{0}$ = enthalpy of micellization *T* = temperature (K)
Tail transfer energy for n-alkyl chains (predicted monotonic behaviour)	${\left({\Delta \mu }_{g}\right)}_{tr-alkyl}/K_{B}T=$ $\left(n–1\right)[{\left({\Delta \mu }_{g}\right)}_{CH2}/K_{B}T]+$ $\left[{\left({\Delta \mu }_{g}\right)}_{CH3}/K_{B}T\right]$	(${\Delta \mu }_{g}{)}_{tr-alkyl}$ = transfer energy for n-alkane chain $K_{B}$ = Boltzmann constant *T* = Temperature ${\left({\Delta \mu }_{g}\right)}_{CH2}$ = tranfer energy for the methylene group ${\left({\Delta \mu }_{g}\right)}_{CH3}$ = tranfer energy for the methyl group
Tail transfer energy for n-alkyl chains (predicted non-monotonic behavior)	${\left({\Delta \mu }_{g}\right)}_{tr}/K_{B}T=[ccc/K_{B}T{]}_{n-1}$ $+\left(1/2\right){\left({\Delta \mu }_{g}\right)}_{CH3}/K_{B}T$	${\left({\Delta \mu }_{g}\right)}_{tr}$ = tranfer energy ${\left({\Delta \mu }_{g}\right)}_{tr-alkyl}$ = transfer energy for imaginary n-alkane chain $K_{B}$ = Boltzmann constant *T* = Temperature ${\left({\Delta \mu }_{g}\right)}_{CH3}$ = tranfer energy for the methyl group
Formation of the Micelle Core-Water Interface	${\left({\Delta \mu }_{g}\right)}_{int}/K_{B}T=\left(\sigma_{agg}/K_{B}T\right)\left(a–a_{0}\right)$, $a_{0}=a_{s}+a_{1}\beta$	${\left({\Delta \mu }_{g}\right)}_{int}$ = energy of interface formation $K_{B}$ = Boltzmann constant *T* = Temperature $\sigma_{agg}$ = interfacial tension between the hydrocarbon core and aqueous solution $a_{0}$ = the interface area that is covered by the surfactant head and counterions that are absorbed at the micelle interface
Surface tension (Eötvös)	$\gamma V^{2/3}=k\left(T_{c}-T\right)$	γ = surface tension *V* = molar volume *k* = $2.1\times 10^{-7}$ $JK^{-1}mol^{2/3}$ $T_{C}$ = critical temperature *T* = temperature
Surface tension (Guggenheim)	$\gamma =\gamma^{0}[1-\left(T/T_{C}\right){]}^{n}$	γ = surface tension $\gamma^{0}$ = constant for each liquid *n* = empirical factor $T_{C}$ = critical temperature *T* = temperature
